# Sarcopenia as a novel prognostic factor in the patients of primary localized gastrointestinal stromal tumor

**DOI:** 10.1186/s12885-022-09278-w

**Published:** 2022-02-17

**Authors:** He Song, Xianhao Xiao, Gang Liu, Jianping Zhou

**Affiliations:** 1 The First Affiliated Hospital of China Medical University, 155 Nanjing Street, Shenyang, Liaoning China; 2 China Medical University, Shenyang, Liaoning China

**Keywords:** Sarcopenia, Gastrointestinal stromal tumor, Skeletal muscle index, Survival

## Abstract

**Background:**

Sarcopenia predicts poor prognosis of a variety of gastrointestinal malignancies. However, there is a lack of study on the association between skeletal muscle index (SMI) and the prognosis of gastrointestinal stromal tumor (GIST). The aim of this study is to develop a novel nomogram based on sarcopenia for GIST patients to predict overall survival (OS).

**Methods:**

SMI was measured by computed tomography scan of 107 patients who underwent resection for primary localized gastrointestinal stromal tumor (GIST). Sarcopenia was defined by cutoff values for SMI as 40.1 cm^2^/m^2^ and 39.8 cm^2^/m^2^ using optimum stratification for males and females respectively. Factors were included in the nomogram were specified by univariate and multiple Cox proportional hazard analysis. Concordance index (C-index) and calibration curves were conducted to measure the discrimination and accuracy of the nomogram. The utility of the nomogram was assessed by the decision curve analysis (DCA).

**Results:**

Twenty-eight (26.2%) of 107 patients were sarcopenic. Sarcopenia was correlated significantly with body mass index, albumin, female sex, resection style, mitotic index, rupture status, survival. Sarcopenia was significantly related to decreased overall survival (*p* = 0.003).The nomogram including sarcopenia status, resection style and mitotic index had an excellent discrimination with C-index 0.794. The calibration curves represented a good accordance between the actual observation and nomogram prediction for overall survival. Decision curve analysis illustrated that the nomogram was helpful in clinic.

**Conclusions:**

We developed a nomogram based on sarcopenia to predict overall survival after resection of GISTs which is an effective and favorable prognostication tool.

## Background

Gastrointestinal stromal tumor (GIST) is the most common mesenchymal neoplasm occurring from the gastrointestinal tract [1]. Worldwide, the prevalence of GIST is 4.3–22 per million per year, with approximately mid 60 s of age and equal gender distribution [2]. GISTs are considered to be with different malignant potential. GIST originating from the Interstitial cells of Cajal or their precursors is able to arise within any part of the GI tract, mostly in stomach (40 ~ 50%) and small intestinal (20 ~ 40%). Primary GISTs of other sites, for instance, colon, esophagus and extra-GI tract sites like omentum or peritoneum are relatively rare. Complete resection to the primary site with clean margin is still the main treatment to localized and resectable GIST.. Before the early 2000s, no effective treatment were availabe, being GIST notoriously resistant to tradiotionaly treatments such as chemotherapy and radiotherapy Identification of the driver mutations in c-KIT and platelet-derived growth factor receptor α (PDGRF-α) further led to the successful targeted therapy of tyrosine kinase inhibitor (TKI)-imatinib [3]. Despite advances in treatment modalities, radical resection with TKI treatment remains mainstay for GIST. Especially, the GIST patients with the intermediate and high risk are recommended for the TKI treatment postoperatively. Risk stratification might differentiate with patients who need TKI treatment and those who do not. Therefore, a more precise and refined stratification system is truly needed to manage postoperative therapeutics.

In 2002, Fletcher [4] firstly developed the predicting system for GIST, presently described as the National Institute of Health (NIH) criteria comprising two factors (mitotic index and tumor size). In 2006, the Armed Forces Institute of Pathology (AFIP) criteria [5] added tumor location to NIH criteria. In 2008, the NIH risk stratification system compared to AFIP was modified to add tumor rupture status. This modified NIH criterion has been widely accepted because it is more easier to apply than the AFIP criteria, and subsequent Memorial Sloan-Kettering Cancer Center (MSKCC) nomogram [6] in 2009 and Joensuu's contour maps [7] in 2012. All the GIST recruited in this cohort were confirmed as the intermediate and high risk category based on modified NIH classifications (tumor size, mitotic rate, location, and rupture).

Sarcopenia is an age-related disorder defined by accelerating depletion in muscle mass and decline in strength and physical performance. Recently, sarcopenia has been regarded as the predictor of poorer disease free survival (DFS) or overall survival (OS) in many malignances [8–10]. Sarcopenia frequently occurs in the disease of chronic disorder such as cancer. In addition, aging could easily induce muscle atrophy[11], and GIST likely occur in the mid 60’s [2]. So, we raised a hypothesis that there might be a relationship between sarcopenia and outcome of GIST.

There are various methods to evaluate the sarcopenia, such as muscle mass, muscle strength and physical performance. Measured value of muscular mass area by a cross-sectional computed tomography (CT) image at the level of the third lumbar vertebra (L3), namely skeletal muscle index (SMI) are generally accepted [12]. Due to SMI based on the preoperative imaging, sarcopenia might be a potential biomarker influencing the prognosis of GIST. However, currently, there were few reports on the role of sarcopenia in patients with GIST.

## Method

### Study design

This study was a single-center retrospective analysis. We evaluated 107 patients with pathologically confirmed GIST with intermediatiate or high risk category who underwent surgical resection in the department of general surgery, the first hospital of China medical university from February 2013 to February 2019. All the patients were followed up postoperatively by telephone interview, outpatient visits and China’s native app-WeChat and all surviving patients were followed up for 6 years. Patient status was due to the time of last follow-up as follows: alive and dead. Survival time was determined as starting from the date of first operation until end of follow-up due to either death or end of data collection. Only “dead” was considered an event in the analysis of overall survival (OS).

This research was approved by Ethics committee of the first hospital of China medical university.

The inclusion criteria were as follows: (1) more than 18 years; (2) abdominal computed tomography (CT) scans available within a month before surgery; (3) primary localized neoplasm; (4) patients received TKI treatment for at least 6 months postoperatively; some patients might have administrated TKIs for second or further line treatment. (5) intermediate and high-risk category according to modified NIH classifications. The exclusion criteria were: (1) distant metastasis;(2) insufficient clinicopathologic data; (3) pretreatment therapy (e.g. preoperative oral administration of imatinib for down stage).

### CT valuation

Every patient of CT-image valuation was performed using ImageJ software.. Skeletal muscle volume was evaluated to use the CT image for all the eligible patients. A transverse CT examination at the third lumbar vertebra (L3) of the single 5 mm-slice was assessed. The muscles in the L3 region—including internal and external obliques, transverse and rectus abdominus, psoas, quadratus lumborum, and erector spinae were analyzed.

L3 skeletal muscle index (L3 SMI, cm^2^/m^2^) is calculated as follows: cross-sectional areas measurements of the muscle (cm^2^) at the L3 divided by the height.

Our own sex-specific cut-off values for the L3 SMI were established at which the survival difference was most significant, we used optimum stratification to define the SMI cut-off value 39.8 cm2/m2 for women and 40.1 cm2/m2 for men. SMI below the defined cutoff value was described as sarcopenia. The method of optimum stratification has been previously described in literature [13] to separate sarcopenic patients and non-sarcopenic patients.

### Statistical analysis

The characteristics of the 2 groups (sarcopenia and non-sarcopenia) were compared. Optimum stratification is applied to find the most significant *p* value by use of the log-rank χ^2^ statistic to define the sex-specific cut-offs associated with overall survival in our cohort. Optimum stratification solves the threshold value of the continuous covariate (skeletal muscle index) which, based on log-rank statistics, best separates sarcopenic patients with non-sarcopenic patients due to end point (death). Univariable analysis was performed using χ^2^ test, the Mann‐Whitney U test for categorical variables and independent-sample* t* test for continuous variables. The Kaplan–Meier method was used to estimate OS. Variables with p < 0.05 in univariable analysis were included in multivariable analysis. A Cox proportional‐hazards regression analysis was performed. The predictive performance of nomogram related to survival was assessed by C-index. The utility of the nomogram was evaluated by decision curve analysis (DCA). *P* value of less than 0.05 was considered significant. All statistical analyses were performed with the Statistical Package for Social Sciences (SPSS) 24.0 for Windows (Chicago, Illinois) and R software (version 3·5·0).

## Results

### Clinical parameters of GIST patients

Among 107 enrolled patients, 28 (26.2%) were classified as the sarcopenia group and 79 (73.4%) into the non-sarcopenia group. The clinical characteristics (Table 1) between the two groups were compared. The mean value of BMI and serum albumin in the non-sarcopenia group was higher than in the sarcopenia group (*P* = 0.001; *P* = 0.007). Significant difference in resection style (*p* = 0.034), mitotic index (*p* = 0.02), rupture status (*p* = 0.007), survival status (*p* = 0.001) were found. Age, hemoglobin, gender, tumor site, tumor size and pathological type were not observed to differ significantly in two groups.

**Table 1:** Comparison of clinicopathological parameters between sarcopenic patients and non-sarcopenic patients

Characteristics	Non-sarcopenia (*n* = 79)	Sarcopenia (*n* = 28)	*P*-value
**Age**	58.5 ± 9.5	58.8 ± 10.2	0.901
**BMI**	23.4 ± 2.9	20.1 ± 2.1	0.001
**Hemoglobin**	119.6 ± 15.9	123.0 ± 19.4	0.355
**Albumin**	41 ± 3.2	40 ± 2.3	0.007
**Gender**			
Male	48	18	0.823
Female	31	10	
**Resection style**			
Complete resection	74	22	0.034
Incomplete resection	5	6	
**Tumor site**			
Stomach	33	10	0.423
Duodenum	27	13	
Colon and rectum	9	4	
Extra GI GIST	10	1	
**Mitotic index**			
less than 5/HPF	30	12	0.02
5–10/HPF	45	10	
more than 10/HPF	4	6	
**Tumor size**			
Less than 5 cm	18	5	
5-10 cm	49	18	0.842
More than 10 cm	12	5	
**Rupture**			
No	75	21	
Yes	4	7	0.007
**Survival**			
Alive	73	16	0.001
Dead	6	12	
**Pathological type**			
Spindle type	52	15	0.35
Epithelioid type	15	9	
Mixed type	12	4	

We also explored the relationship between sarcopenia and the degree of postoperative complications according to the Clavien–Dindo classification (Table 2). Complications occurred in 46 cases. The majority of complications were concentrated in grade I and grade II, which accounted for 87% (40/46). Five patients were performed by second operation or interventional therapy due to anastomotic leakage and abdominal infection, which were classified as complication of grade III. One patient occurred heart failure of grade IV complications. None died in the perioperative period. The incidence of complications differed significantly in the two groups (*p* = 0.001).

**Table 2:** Comparison of postoperative complications between sarcopenic patients and non-sarcopenic patients

Charateritics	Non-sarcopenia (*n* = 79)	Sarcopenia (*n* = 28)	*P*-value
**Grade I**			
Uroschesis	4	2	
Incisional infection	3	3	
**Grade II**			
Pulmonary atelectasis	3	4	
Incisional infection	2	4	
Adhesive intestine obstruction	3	2	
Abdominal infection	4	1	
Cardiac dysfuction	2	0	
Incisional hernia	1	2	
**Grade III**			
Anastomotic leakage	1	1	
Abdominal infection	2	1	
**Grade IV**			
Heart failure	1	0	
**Grade V**			
NA	0	0	
**Total, %**	26(32.9%)	20(71.4%)	0.001
**NA not available**			

By univariate analysis, sarcopenia (*p* = 0.003), resection style (*p* = 0.001) and mitotic index (*p* = 0.005) were significant predictors of OS. They were also further conducted in the multivariate analysis. sarcopenia (*p* = 0.016), resection style (*p* = 0.036) and mitotic index (*p* = 0.044) were still identified as the independent prognostic factor for OS in GIST patients. (shown in the Table 3).

**Table 3:** Univariate and multivariate analysis for overall survival

Variable	Univariare	analysis	Multivariate	analysis
	OS		OS	
	OR(95%CI)	*P*-Value	OR(95%CI)	*P*-Value
Age				
< 65	Reference	0.387		
≥ 65	0.485(0.129–1.816)			
Gender				
Female	Reference	1.000		
Male	0.971(0.343–2.747)			
BMI		0.346		
< 18.5	Reference			
18.5 ≤ ≤ 23.9	2.377(0.277–20.374)	0.676		
> 24	1.000(0.092–10.865)	1.000		
Sarcopenia				
No	Reference	0.003	Reference	0.016
Yes	4.475(1.689–11.930)		3.488(1.261–9.639)	
HB(g/L)		0.183		
< 120	Reference			
≥ 120	0.535(0.216–1.318)			
Albumin				
< 40	Reference	0.151		
> 40	0.567(0.231–1.380)			
Resection				
Complete	Reference	0.001	Reference	0.036
Incomplete	5.223(2.013–13.565)		2.482(1.887–7.025)	
Site				
Stomach	Reference	0.061		
Duedenum + intestine	1.721(0.448–6.611)	0.511		
Colon and rectum	8.357(1.866–37.428)	0.007		
Extra GI GIST	2.167(0.342–13.720)	0.590		
Pathological type				
Spindle	Reference	0.486		
Epithelioid	1.500(0.455–4.943)	0.530		
Mixed	1.315(0.317–5.463)	0.708		
Size(cm)				
< 5	Reference	0.012		
5–10	2.437(0.662–3.213)	0.545		
> 10	11.351(0.718–12.125)	0.232		
Mitotic Index				
< 5	Reference	0.005	Reference	0.044
5–10	1.617(0.452–5.782)	0.545	4.950(0.863–28.571)	
> 10	14.250(2.788–72.845)	0.002	2.089(1.021–4.225)	

**Fig. 1:**
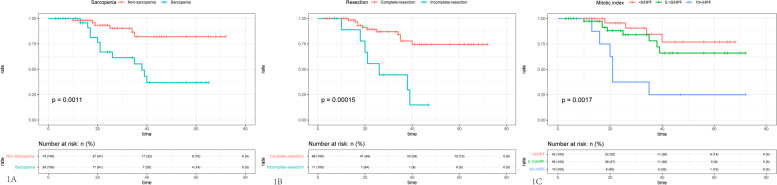
Kaplan‐Meier curves of overall survival according to (**A**) sarcopenia status, (**B**) resection style and (**C**) mitotic index. Horizontal axis is calculated by month

At the time of final follow‐up (Feb, 2019), 18 of 107 patients (16.8%) were dead, and the median follow‐up for was 25.4 months. The median overall survival of patients with sarcopenia defined by SMI was significantly lower (40.6 months; 95% CI, 31.6‐49.6) than those with normal skeletal muscles (63.8 months; 95% CI, 57.6‐69.8; *P* = 0.0011) (shown in the Fig. 1A). The patients undergoing incomplete resection represented the poorer survival compared to the patients with complete resection (*p* = 0.00015, shown in the Fig. 1B). The patients with high mitotic index (> 10HPF) represented significantly worst survival (*p* = 0.0017, shown in the Fig. 1C).

### Establishment of nomogram

**Fig. 2:**
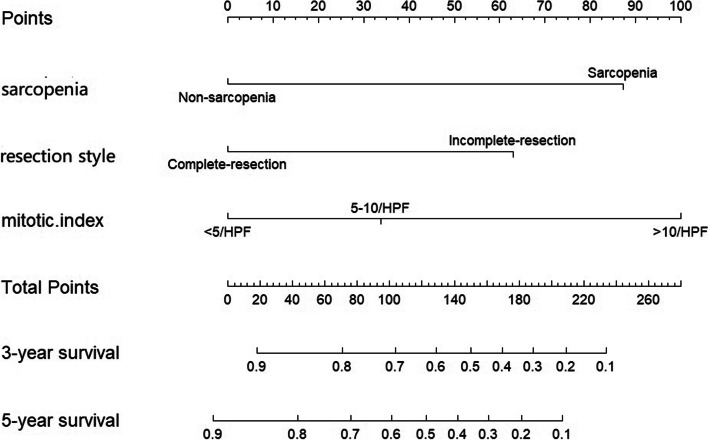
Nomogram for predicting GIST-related survival with sarcopenia status, resection style and mitotic index. Note: The probability of each variable was added to converted into total score, and a vertical line was drawn on the total score to achieve the related probability of death

In the multivariable COX proportion hazard regression models, 3 factors including sarcopenia status, resection style and mitotic index were significantly associated with mortality of GIST. Therefore, nomogram of three factors was established (shown in the Fig. 2) to predict the probability of 3 year- and 5 year- OS.

**Fig. 3:**
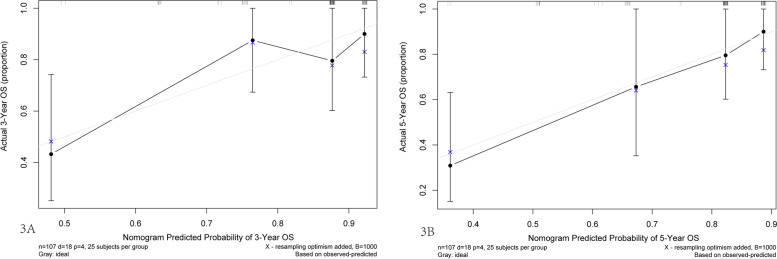
Calibration curves of the prognostic nomogram for 3-year overall survival (**A**), 5-year overall survival (**B**). The Y-axis indicates the observed overall survival of GIST while the X-axis indicates the estimated overall survival. The solid line demonstrates the ideal reference line that predicted GIST survival associated with the actual outcome whereas the dashed line demonstrates the prediction of nomogram. The closer alignment with the solid line represents the better performance is acquired

The associated concordance index of nomogram (c-index) was 0.794 (95% CI 0.747–0.841), which indicated that 79.4% of the probability of individual mortality would be correctly predicted by the nomogram model. The calibration curves represented high consistency in the prognostic value of 3 year- and 5 year-OS (Fig. 3A + B).

### Clinical use of DCA curve analysis

**Fig. 4:**
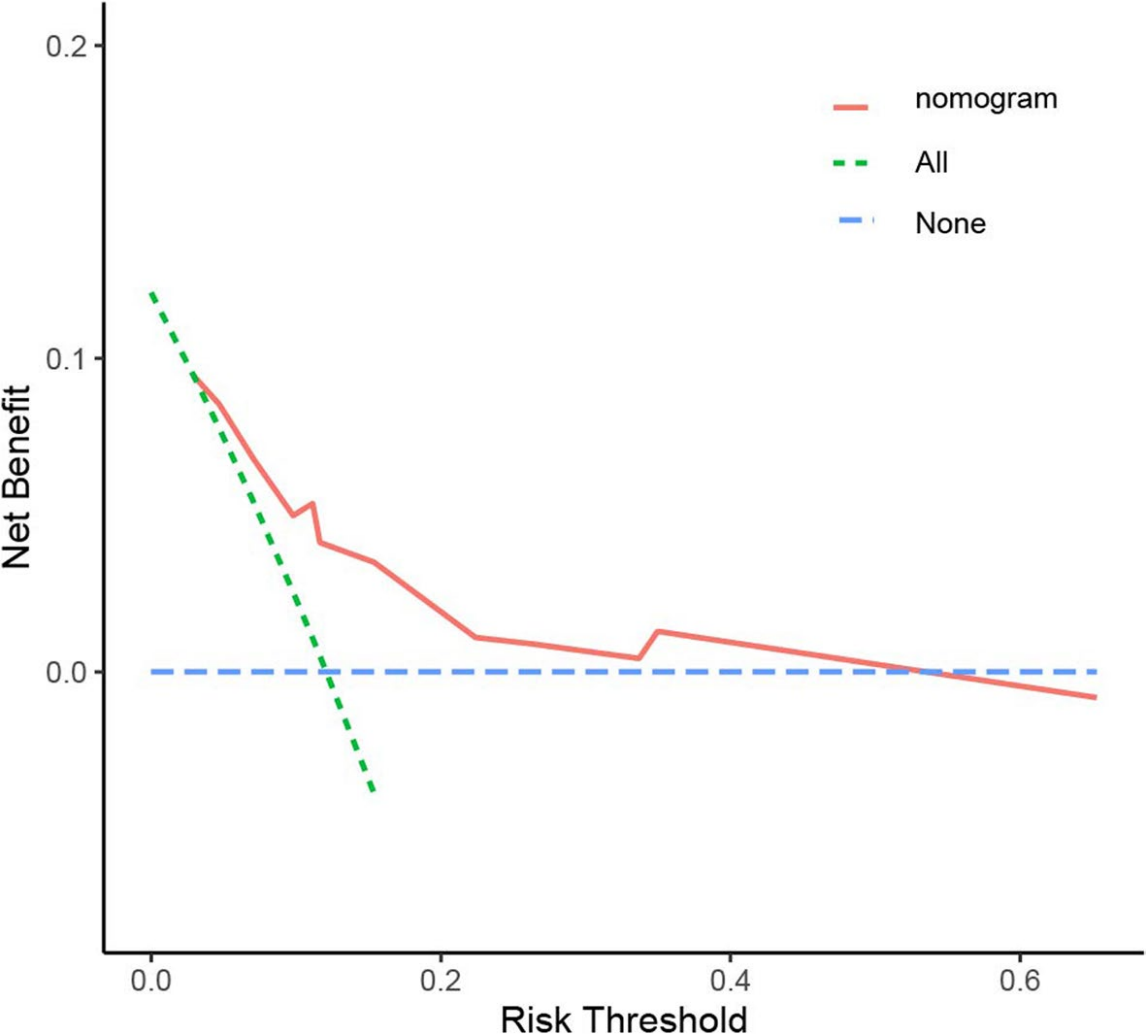
The Decision Curves Analysis curve of the predictive nomogram including three factors (sarcopenia status, resection style, mitotic index). The horizontal axis represents the threshold value, which is the where the expected benefit of treatment was equal to the expected benefit of avoiding treatment and the vertical axis represents adding up the true positive results and subtracting the false positive results. The nomogram (red line) has the high value due to the larger net benefit

The decision curve analysis (shown in the Fig. 4) elucidated that the nomogram was feasible to make clinical valuable decision.

## Discussion

Our research showed a comprehensive analysis from clinicopathological data and CT scans to explore the survival outcomes in the Chinese population with GIST following surgery. Preoperative sarcopenia defined by SMI was associated with poor survival. CT has a high degree of validity in assessing body composition and is regarded as the gold standard method for estimating muscle [10, 14, 15].To the best of our knowledge, this is the first research indicating that impaired effects of sarcopenia for the survival in patients with GIST worldwide.

Sarcopenia is a syndrome affecting innumerable people with cancers and is independent predictor of detrimental outcomes such as physical disability, poor quality of life, and reduced survival [16].However, the clinical definition of SMI remains inconclusive, the most widely used definitions were defined by Prado [13] in the western people. However, these definitions might not be applicable to Chinese GIST patients because BMI and physique differs greatly between eastern and western populations. Therefore, we used optimum stratification analysis to define the SMI cutoff. The incidence of sarcopenia with GIST patients was 26.2% (28/107) in our study is consistent with the previous study [17] with sarcopenic morbidity of 38.7% in the GIST patients.

Sarcopenic patients represented lower BMI and serum albumin. These two factors were associated with sarcopenia but not with the survival. Sarcopenia is considered to be a better predictive tool than BMI and albumin for survival. Albumin is a negative acute-phase protein that decreases in concentration with ongoing systemic inflammation, poor health, and malnutrition which lead to the decreased skeletal muscle mass [18], hence, lower albumin might be associated with low-SMI value to reflect the sarcopenic condition. This study is conformable with previous findings in other cancers [19, 20].

The high efficient predictive tool is indispensable for GIST patients. At present, a few influential predictive models for the prognosis of GIST have been designed. Miettinen [5] proposed that tumor size, mitotic rate, and tumor site can accurately predict the risk of GIST patients. Gold developed a nomogram for the recurrence free survival of patients with GIST patients after complete resections [6]. This study established a new nomogram including resection style, mitotic index and sarcopenia. To prove the clinical validity, we evaluated whether the nomogram-assisted decisions would be beneficial to patient outcomes or not. The novel method of decision curve analysis demonstrated that if the threshold probability of a patient or doctor is 40%, then probably 7.5 persons would benefit without detriment of others. Our nomogram also represent the reliable performance with high c-index of 0.794. Our nomogram did not include other important clinical factors, for instance, the tumor size, tumor site and presence of rupture which have been reported to be correlated with the mortality of GIST patients [21–23]. The possible reason might be some patient records affecting the data bias was excluded for insufficient clinical data. In the future study, we need the more clinical samples and validation set to prove our nomogram.

To reinforce the precision of tumor biological behavior prediction by only depending on resection style and mitotic counts, we integrated the new parameter sarcopenia by measurement of SMI into our nomogram. Preoperative and postoperative SMI are of equal importance. An interesting study [17] showed 63.6% of initially sarcopenic GIST patients became non-sarcopenic after 6 months of imatinib. This reversal might be explained by the drug’s anti-tumor activity. Hence, clinicians should persist in nutritional guidance for the whole management of GIST patients. Meanwhile, patients are encouraged to exercise and receive appropriate nutritional treatment for muscle protein synthesis against sarcopenia [11, 24].

This study had several limitations. First, according to the European Working Group on Sarcopenia in Older People (EWGSOP), sarcopenia should be measured by the parameters of muscle mass, muscle strength and physical performance [25]. We regarded only muscle mass as the definition of sarcopenia due to the retrospective design, and prospective study is further needed including more assessment tools for sarcopenia. Second, merely 107 patients were enrolled due to insufficient available clinical data and low incidence of GIST. Third, this was a single-institution study of small sample, and whether the results are feasible for other patient sets which needs further internal and external validation. Nevertheless, to best of our acknowledgement, this is the first study to establish a predicting model based on the preoperative sarcopenia of GISTs patients. More variables, for instance, inflammation index and gene detection could be incorporated in future.

## Conclusion

In summary, we conducted a comprehensive analysis of preoperative sarcopenia associated with survival prognosis and established a new nomogram accurately predicting 3- and 5- survival for GIST patients. Thus, this study might be helpful for the physician to make better clinical evaluation.

## Data Availability

The datasets during and/or analyed during the current study available from the corresponding author on reasonable request.

## Abbreviations

GIST: Gastrointestinal stromal tumor,
OS: Overall survival
